# Analysis of Trends and Influencing Factors of Cytokine-Adsorbing Therapies: A Nationwide Ecological Study in Japan

**DOI:** 10.7759/cureus.73489

**Published:** 2024-11-11

**Authors:** Shohei Ono, Keiki Shimizu

**Affiliations:** 1 Department of Anesthesiology and Critical Care, Jichi Medical University, Saitama Medical Center, Saitama, JPN; 2 Department of Emergency and Critical Care Medicine, Tokyo Metropolitan Tama Medical Center, Tokyo, JPN

**Keywords:** an69st, continuous renal replacement therapy, ecological study, hemoadsorption, ndb open data, pmx-dhp, sepxiris○r

## Abstract

Background: Cytokine-adsorption therapy has garnered attention as a potential treatment for conditions such as sepsis, although supporting evidence remains limited. Consequently, its utilization is expected to vary significantly across regions. To date, no ecological studies have investigated this regional heterogeneity.

Objective: This study aimed to examine temporal trends in the use of continuous renal replacement therapy (CRRT) with cytokine-adsorbing hemofilters and polymyxin-B immobilized fiber-direct hemoperfusion (PMX-DHP), as well as the spatial distribution of both across Japan's 47 prefectures.

Methods: This ecological study analyzed National Database (NDB) open data. A longitudinal analysis from 2016 to 2022 assessed temporal trends in the use of adsorption membranes. A cross-sectional analysis of the 2022 data utilized Moran's I statistic to evaluate the spatial autocorrelation of adsorption therapy. To examine the relationship between the two types of adsorption therapy, we calculated the Pearson correlation coefficient and conducted a multivariate analysis.

Results: The longitudinal analysis revealed no significant change in the proportion of cytokine-adsorbing hemofilter use, while PMX-DHP use showed a decreasing trend over the seven-year period. Cross-sectional analysis indicated spatial autocorrelation for both PMX-DHP (Moran's I: 0.34, P < 0.001) and cytokine-adsorption filter use (Moran's I: 0.24, P < 0.001). Univariate analysis (R = -0.29, P = 0.0453) and multivariate analysis (estimated coefficient: 1.27, 95% CI: 0.06-2.49, P = 0.045) demonstrated that higher usage rates of cytokine-adsorbing blood filters were associated with higher PMX-DHP usage rates.

Conclusions: This study identified a declining trend in PMX-DHP use and an association between PMX-DHP and cytokine-adsorbing hemofilter utilization. These findings suggest that physicians' preferences and perceptions regarding cytokine-adsorption therapy may influence its use. Further research with individual patient data is warranted to confirm these findings.

## Introduction

In recent years, various forms of cytokine-adsorption therapy have been implemented, with an expanding range of clinical indications [1-11]. Among these therapies, polymyxin-B immobilized fiber-direct hemoperfusion (PMX-DHP), CytoSorb®, and continuous renal replacement therapy (CRRT) with cytokine-adsorption hemofilter are the most commonly used. The efficacy of cytokine-adsorption hemofilters for CRRT has been demonstrated in observational studies [12-15]. However, randomized controlled trials (RCTs) have been small in scale and have shown reductions only in inflammatory substances, not in mortality [4,12-14,16]. Recent RCTs involving PMX-DHP have yielded negative results [8-10]. In 2016, the Surviving Sepsis Campaign Guidelines (SSCG) did not recommend PMX-DHP [17]. However, following the results of the 2018 EUPHRATES study [8], the SSCG revised its recommendation in 2021 to "suggest against using polymyxin-B hemoperfusion" (weak recommendation; low quality of evidence) [18]. Despite the limited evidence supporting cytokine-adsorption therapy, new adsorption treatments are being introduced into clinical practice. Supady et al. indicated in their review that seven cytokine-adsorption therapies and 28 RCTs were underway as of 2022 [19]. They noted, "Considering the current lack of evidence for a benefit of hemoadsorption for the treatment of severe inflammation, sepsis, liver failure, and rhabdomyolysis, its routine use in everyday clinical practice cannot be justified."

A notable contradiction exists between the limited evidence supporting adsorption therapy and the reality of its expanding indications, often determined by the discretion of the treating physician. Treatment methods influenced by individual perspectives may contribute to regional variability; however, no ecological study on adsorption therapy has been conducted to date. Ecological research, which uses regions as the unit of analysis, can clarify spatial distributions and identify region-specific factors [20,21]. While causal inference is challenging due to the cross-sectional nature of such research, it can reveal prevalent interpretation trends in specific regions and highlight the impact of information literacy on clinical decision-making [22-25].

Therefore, we conducted an ecological study using the NDB (National Database of Health Insurance Claims and Specific Health Checkups of Japan) open data [26] to clarify the trends in cytokine-adsorption therapies and the factors influencing them.

## Materials and methods

Study design

This ecological study utilizes two open datasets to investigate cytokine-adsorption therapy across Japan's 47 prefectures. The first dataset is the NDB open data [26], a comprehensive nationwide administrative claims database maintained by the Ministry of Health, Labor and Welfare (MHLW), which includes health insurance claims and medical examination records. The second dataset is e-Stat, managed by the Statistics Bureau of the Ministry of Internal Affairs and Communications, which aggregates over 700 datasets from various government ministries and agencies, enabling detailed data searches and outputs [27]. As these datasets do not contain personal information, anonymization was not required.

The study consists of two parts: (i) a longitudinal analysis of NDB open data from the second period (April 1, 2016, to March 31, 2017) to the ninth period (April 1, 2022, to March 31, 2023) to assess trends in PMX-DHP use and the proportion of cytokine-adsorbing hemofilter use; and (ii) a cross-sectional analysis using the ninth NDB open data update along with other open data on a prefecture-by-prefecture basis. In the longitudinal analysis, we excluded the first part of the update due to differences in data availability. This study adhered to the principles of the Declaration of Helsinki. All data used in the analysis were anonymized and did not contain individual patient information. The study protocol was approved by the ethics committee of Tokyo Metropolitan Tama General Medical Center (approval no. 6-122). The study's findings have been reported in accordance with the STROBE (strengthening the reporting of observational studies in epidemiology).

Data collection

The study included all 47 prefectures in Japan. We utilized reports on specific medical materials (annual consumption of normal hemofilters and cytokine-adsorbing hemofilters for CRRT and PMX-DHP) and medical procedures (annual number of PMX-DHP procedures performed) by prefecture from the NDB Open Data. While the usage of PMX-DHP was analyzed as is, the amount of CRRT was expected to influence the amount of adsorbent membrane utilized. Therefore, it was standardized using the following formula: cytokine-adsorbing membrane (AN69ST) use/general membrane use. Additionally, we referenced e-Stat to gather relevant data: population, the proportion of males and proportion of individuals aged 65 and over, the number of physicians, the proportion of intensivists, nephrologists, emergency physicians, areas, and population density. The data on specific medical materials, procedures, and number of physicians were converted to per 100,000 persons.

Statistics

We utilized the second and ninth NDB open data to display trends in the use of cytokine-adsorbing hemofilter use (AN69ST use/total hemofilter use) and PMX-DHP through boxplots. We subsequently performed a cross-sectional analysis using the ninth NDB open dataset. We calculated Moran's I statistics for cytokine-adsorption therapy to assess regional transmissibility. Moran's I statistic is a method for assessing the presence of spatial autocorrelation: a high correlation coefficient indicates similar distributions among neighboring regions, a coefficient near zero signifies a random distribution, and a negative coefficient reveals a "marbled" pattern [28-30]. By smoothing the count of adsorption therapies through a hierarchical Bayesian model, we addressed potential disparities arising from differences in prefectural sizes. Subsequently, we divided the ninth NDB open data for the 47 prefectures into two groups based on the median values of cytokine-adsorbing hemofilter use, categorizing them as high and low use. Scatter plots were created to visualize the relationship between each measurement and the proportion of cytokine-adsorbing hemofilter use as a continuous variable. Pearson's correlation coefficients were calculated to assess these relationships.

We performed a multivariable analysis to examine the relationship between the utilization rates of cytokine-adsorption hemofilters and PMX-DHP. Confounding factors were selected using a directed acyclic graph (DAG), focusing on prefectural characteristics.

In the treatment and specific medical material data from the NDB open data, extremely small numbers of cells (fewer than 20) are reported as missing values. However, the total number of missing values can be obtained. Therefore, we used the mean imputation method, dividing the total number of missing values by the number of missing cells for each variable, and included this in the analysis.

Data were expressed as means and standard deviations where appropriate. The two-tailed significance level was set at p=0.05, and all statistical analyses were performed using R-4.3.1 (R Foundation for Statistical Computing, Vienna, Austria).

## Results

**Figure 1:**
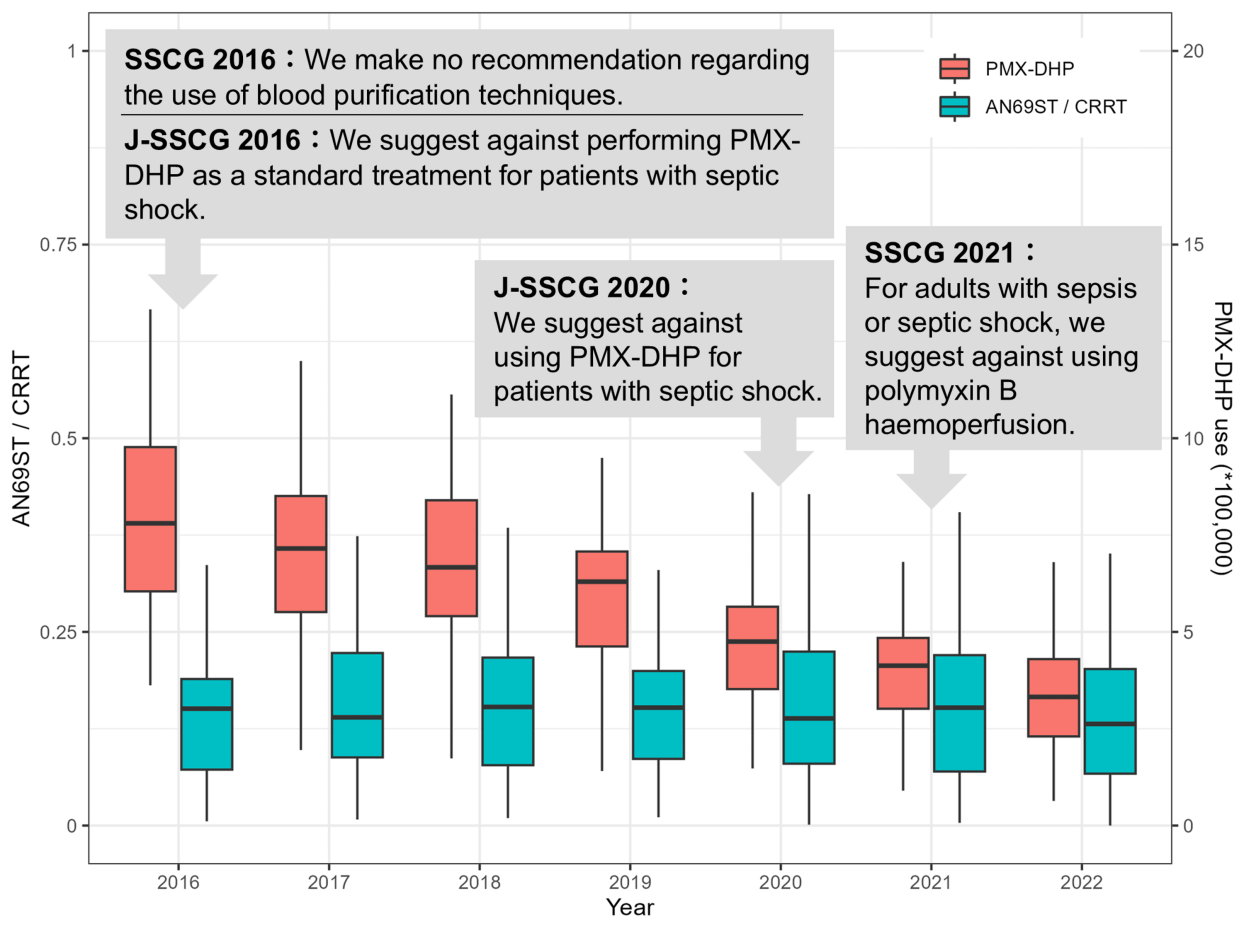
Boxplots of the temporal trend of cytokine-adsorption therapies The orange box indicates the amount of PMX-DHP use, and the green box indicates the proportion of cytokine-adsorbing filter use (AN69ST use/general filter use). SSCG: Surviving Sepsis Campaign International Guidelines for Management of Sepsis and Septic Shock; J-SSCG: The Japanese Clinical Practice Guidelines for Management of Sepsis and Septic Shock; PMX-DHP: polymyxin-B immobilized fiber-direct hemoperfusion; AN69ST: acrylonitrile-co-methallyl sulfonate surface-treated; CRRT: continuous renal replacement therapy

We displayed the temporal trend of the annual proportion of cytokine-adsorbing hemofilter use (AN69ST use/total hemofilter use) and PMX-DHP from 2016 to 2022 in Figure 1. The proportion of cytokine-adsorbing hemofilter use neither increased nor decreased, while PMX-DHP use decreased.

Using the ninth NDB open data, we performed cross-sectional analyses, dividing the data for the 47 prefectures into two groups based on the median value of the proportion of cytokine-adsorbing hemofilter use (13.1%). There was a trend toward a larger population, larger areas, fewer physicians, and less CRRT in the high-use group; however, none of these differences were statistically significant (Table 1).

**Table 1:** Baseline of 47 prefectures in 2022 * indicates multiplication by 1,000 All data are expressed as means (standard deviation) CRRT: continuous renal replacement therapy; PMX-DHP: polymyxin-B immobilized fiber-direct hemoperfusion; AN69ST: acrylonitrile-co-methallyl sulfonate surface-treated

Baseline Data	Low Adsorption Membrane Uses (N=23)	High Adsorption Membrane Uses (N=24)	P-value
Prefecture characteristics			
Population (*1,000)	2081 (2048)	3262 (3304)	0.15
Mean age (yrs)	49 (1.8)	49 (1.9)	0.855
Area (km^2^)	5916 (3039)	10079 (15995)	0.226
Density population (/km^2^)	509 (933)	799 (1455)	0.421
Aged over 65 (%)	31 (2.9)	31 (3.4)	0.954
Men (%)	48 (0.8)	48 (1.1)	0.528
Number of physicians			
Number of physician (/100,000)	271 (32.3)	252 (47)	0.109
Nephrologist (%)	1.54 (0.3)	1.57 (0.6)	0.855
Emergency physician (%)	1.18 (0.4)	1.10 (0.3)	0.418
Intensivist (%)	0.28 (0.2)	0.23 (0.1)	0.284
Treatment			
PMX-DHP (/100,000)	3.37 (1.8)	3.60 (2.2)	0.7
CRRT (/100,000 person)	86 (36)	70 (25)	0.067
AN69ST (/100,000 person)	6.31 (4.3)	19.39 (9.0)	<0.001
Proportion of cytokine-adsorbing hemofilter (AN69ST use/total hemofilter use)	0.07 (0.1)	0.22 (0.1)	<0.001

**Figure 2:**
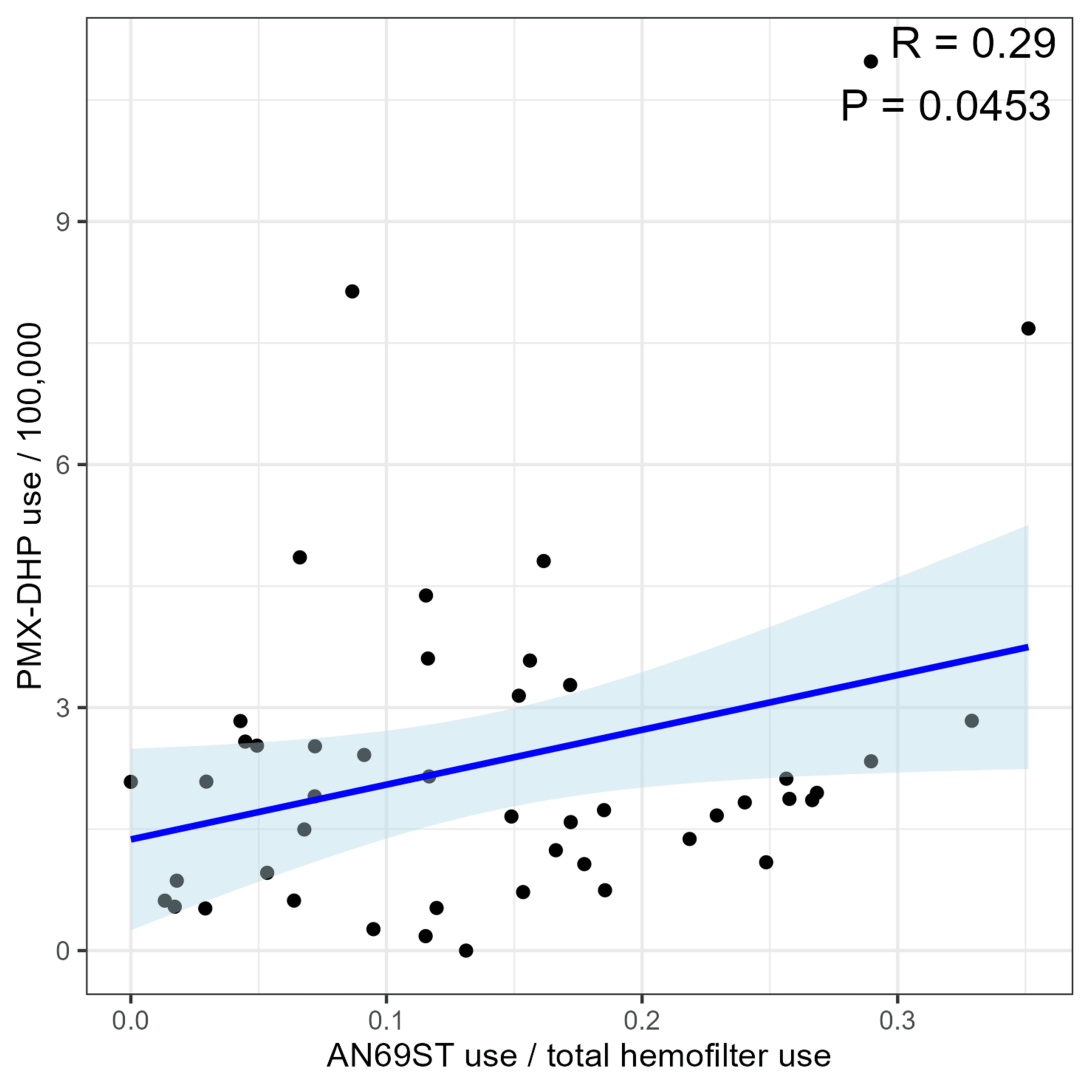
Scatterplot of PMX-DHP use and the proportion of cytokine-adsorbing hemofilter used for CRRT (AN69ST use/total hemofilter use) PMX-DHP: polymyxin-B immobilized fiber-direct hemoperfusion; CRRT: continuous renal replacement therapy; AN69ST: acrylonitrile-co-methallyl sulfonate surface-treated

**Figure 3:**
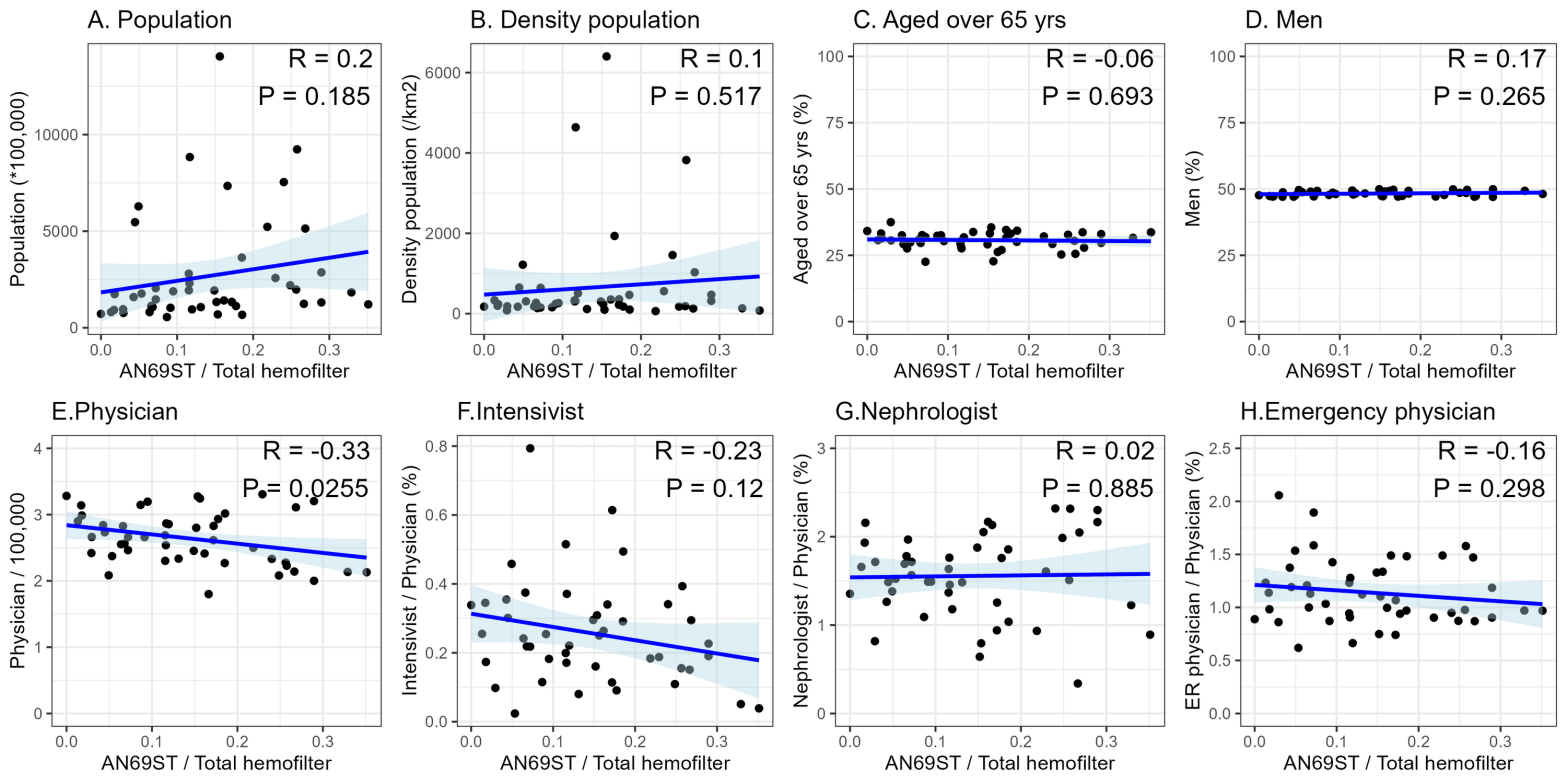
Scatterplot of the proportion of cytokine-adsorbing hemofilter used for CRRT (AN69ST use/total hemofilter use) and each variable * indicates multiplication by 1,000 CRRT: continuous renal replacement therapy; AN69ST: acrylonitrile-co-methallyl sulfonate surface-treated

Next, we conducted univariable and multivariable analyses with the proportion of cytokine-adsorbing hemofiltration use as the dependent variable. Explanatory variables included PMX-DHP, CRRT, population, and number of physicians, selected based on a DAG. We presented scatterplots showing the relationship between each measurement and the proportion of cytokine-adsorbing hemofilter use as a continuous variable (Figures 2, 3). A higher proportion of cytokine-adsorbing hemofilter use was related to a larger population (R=-0.2, P=0.185), fewer physicians (R=-0.34, P=0.0208), and more PMX-DHP use (R=-0.29, P=0.0453). Correlation coefficients for CRRT, population density, and the proportion of those aged over 65 years were all less than 0.1, and no clear trend could be observed. Statistically significant differences were observed only for PMX-DHP use and the number of physicians.

**Figure 4:**
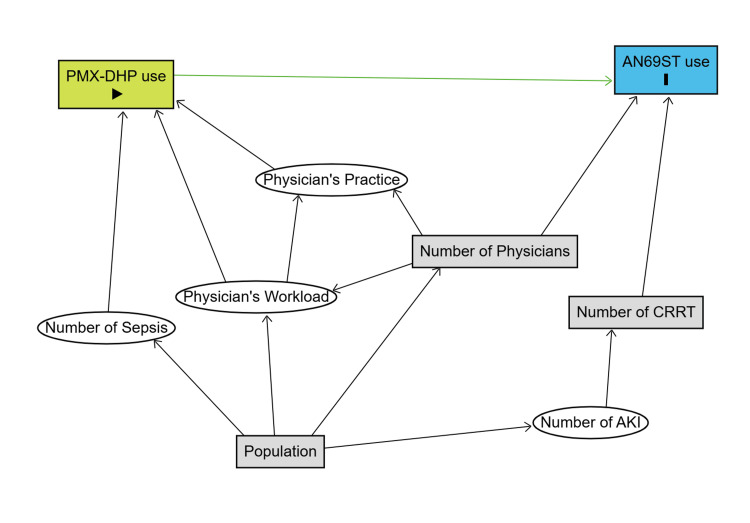
Directed acyclic graph A directed acyclic graph (DAG) is a tool for organizing hypotheses about causal relationships between variables. This graph illustrates that potential confounding can be controlled by adjusting for CRRT, population size, and the number of physicians. CRRT: continuous renal replacement therapy Image created by the authors using DAGitty (https://www.dagitty.net/dags.html)

We then performed univariable and multivariable analyses. We set PMX-DHP, CRRT, population, and number of physicians as explanatory variables and the proportion of cytokine-adsorbing hemofilter use as the objective variable. The estimated coefficients, 95% confidence intervals (CI), and P-values for the explanatory variables in the multivariable analysis are presented in Table 2. Statistically significant associations were observed for PMX-DHP use (crude coefficient: 1.40, 95% CI: 0.24 to 2.56, P-value: 0.023; adjusted coefficient: 1.27, 95% CI: 0.06 to 2.49, P-value: 0.045) and the number of physicians (crude coefficient: -0.08, 95% CI: -0.14 to -0.02, P-value: 0.016; adjusted coefficient: -0.08, 95% CI: -0.14 to -0.01, P-value: 0.021). Other variables were not statistically significant. We used a DAG to select the explanatory variables for adjustment in the multivariate analysis (Figure 4).

**Table 2:** Results of the multivariable analysis with the proportion of cytokine-adsorbing hemofilter use (AN69ST use/total hemofilter use) as the objective variable We showed all estimated coefficients and 95% confidence intervals, both per 100,000 population. CI: confidence interval; CRRT: continuous renal replacement therapy; PMX-DHP: polymyxin-B immobilized fiber-direct hemoperfusion

Variables	Unadjusted		Adjusted	
	Estimated coefficients (95% CI)	P-value	Estimated coefficients (95% CI)	P-value
PMX-DHP	1.40 (0.24 to 2.56)	0.023	1.27 (0.06 to 2.49)	0.045
CRRT	0.00 (-0.05 to 0.06)	0.889	0.01 (-0.06 to 0.07)	0.877
Population	0.001 (0 to 0.001)	0.232	0.001 (0 to 0.002)	0.185
Physician	-0.08 (-0.14 to -0.02)	0.016	-0.08 (-0.14 to -0.01)	0.021

Finally, we included all variables listed in Table 1 in a multivariable model using a stepwise approach for sensitivity analysis (Table 3). The results showed that the number of physicians lost statistical significance, but the estimated coefficient for PMX-DHP use remained statistically significant, indicating the robustness of the main analysis.

**Table 3:** Results of multivariable analysis by stepwise approach We showed all estimated coefficients and 95% confidence intervals, both per 100,000 population. Akaike information criterion of best model: -94.67. CI: confidence interval; CRRT: continuous renal replacement therapy; PMX-DHP: polymyxin-B immobilized fiber-direct hemoperfusion

Variables	Estimated Coefficients (95% CI)	P-value
PMX-DHP	1.48 (0.35 to 2.6)	0.014
Population	0.001 (0 to 0.002)	0.087
Physician	-0.08 (-0.14 to -0.02)	0.011
Aged over 65	0.65 (-0.39 to 1.70)	0.226

## Discussion

This ecological study aimed to identify trends and factors associated with cytokine-adsorption therapies in Japan. Longitudinal analysis showed no clear trend of increase or decrease in cytokine-adsorbing hemofilter use; however, PMX-DHP use showed a decreasing trend over a seven-year period. The cross-sectional analysis revealed that the use of PMX-DHP and cytokine-adsorbing hemofilter exhibited spatial autocorrelation, indicating a geographic association between areas where these interventions were utilized.

The 2009 EUPHAS study demonstrated a reduction in mortality with PMX-DHP treatment [11]; however, subsequent findings from three RCTs have refuted its efficacy [8-10]. Although effective subgroups have been explored in recent years (NCT03901807), the impact of the change in recommendation by the SSCG is significant [17,18]. On the other hand, only observational studies or small RCTs were conducted on cytokine-adsorbing hemofilter use for CRRT, and the SSCG did not mention this treatment. Therefore, there was little information for physicians to make informed decisions. The longitudinal changes in PMX-DHP and cytokine-adsorbing hemofilter use observed in our study may be influenced by regional variations in information literacy thresholds. In addition, the implementation of cytokine-adsorbing hemofilters involves lower invasiveness than PMX-DHP, as it merely requires the choice of the hemofilter. The low disadvantage of this treatment might motivate its selection, even if the scientific evidence is limited.

The association of these therapies was observed in cross-sectional analyses. Although both treatments share similarities in cytokine-adsorption, they also have differences: PMX-DHP is primarily used for stabilizing kinetics, whereas CRRT with cytokine-adsorbing hemofilters is used for acute kidney injury patients with hemodynamic instability requiring dialysis. The association of these therapies suggests that physicians may consider them as a single category of cytokine-adsorption therapy, and study results for one treatment might be extrapolated to the other. Additionally, since both therapies involve blood access catheters, they might be performed concurrently. However, the results of PMX-DHP studies [1-3,15] cannot necessarily be applied to CRRT with cytokine-adsorbing hemofilters. The results of this study may provide insights into physicians' interpretations and preferences.

Our study has two strengths. First, it is the first to evaluate the factors associated with cytokine-adsorbing hemofilter use in CRRT, reflecting physicians' thought processes and preferences. Second, it was an all-Japan survey, providing highly comprehensive results. In summary, this study offers valuable insights into the use and perception of cytokine-adsorption therapies in Japan. Despite the limited evidence base, the lower invasiveness and shared characteristics with other cytokine-adsorption treatments influence their use. Future studies should aim to include individual patient data and consider broader geographical contexts to enhance the robustness and generalizability of the findings.

Limitation

Our study also has two limitations. First, the small sample size due to the prefecture-based analysis and the limited number of measured items did not allow for the adjustment of various confounders. Individual patient data would be desirable for more robust analysis and causal inferences. Second, there is no external validity to regions outside of Japan. In Japan, the flow rate of CRRT is often less than in previous studies due to upper insurance limits for dialysate.

## Conclusions

This ecological study identified trends and factors associated with cytokine-adsorption therapies in Japan. The longitudinal analysis showed no significant change in cytokine-adsorbing hemofilter use, while PMX-DHP use decreased over seven years. Despite limited evidence supporting the efficacy of cytokine-adsorption therapy, their use continues, likely influenced by clinician preferences and perceptions. Our survey results indicated that CRRT with cytokine-adsorption hemofilters and PMX-DHP tend to be utilized within the same regions, suggesting that region-specific information literacy may influence these treatment choices. This study could provide insights into physicians' interpretations and the factors influencing the use of cytokine-absorption therapies.
